# Sources and Toxicity of Mercury in the San Francisco Bay Area, Spanning California and Beyond

**DOI:** 10.1155/2020/8184614

**Published:** 2020-09-24

**Authors:** Mietek Kolipinski, Mani Subramanian, Kristina Kristen, Steven Borish, Stacy Ditta

**Affiliations:** ^1^Department of Natural Sciences and Mathematics, Dominican University of California, San Rafael, CA 94901, USA; ^2^Department of Human Development and Women's Studies, California State University East Bay, Hayward, CA 94542, USA

## Abstract

This report synthesizes and evaluates published scientific literature on the environmental occurrence and biomagnification of mercury with emphasis on the San Francisco Bay Area (SFBA), California. Mercury forms various compounds, well known for their toxicity in humans and environmental ecosystems. Elemental mercury is transported and distributed by air, water, and sediments. Through the metabolic processes of algae and bacteria, mercury is converted into organic compounds, such as methylmercury (MeHg), which then bioaccumulates up through trophic levels. In fish, it is found primarily in skeletal muscle, while in humans, the primary target organs are the brain and kidneys. Health concerns exist regarding bioaccumulation of mercury in humans. This paper reviews the known anthropogenic sources of mercury contamination, including atmospheric deposition through aerial transport from coal burning power plants, cement production, and residual contaminants of mercury from gold mining, as well as mercury-containing waste from silver amalgams emitted from dental offices into waterways. Although tools exist for measuring mercury levels in hair, breast milk, urine, blood, and feces in humans, current diagnostic tools are inadequate in measuring total mercury load, including deposited mercury in tissues. Additionally, insufficient attention is being paid to potential synergistic impacts of mercury interaction with multipliers such as lead, cadmium, and aluminum. We provide specific data on methylmercury concentrations at different trophic levels, followed by recommendations for reducing the level of mercury in the SFBA in order to protect the health of humans and other species.

## 1. Introduction

Mercury, atomic symbol, Hg (Latin, *Hydrargyrium*), is one of the most highly toxic, nonradioactive elements [1]. The three primary forms of mercury are elemental mercury (Hg^0^), inorganic mercury (Hg^2+^), and organic mercury compounds (MeHg and EtHg), all of which are deleterious to humans and other taxa. Mercury exposure at toxic levels most commonly affects neurologic, renal, and gastrointestinal systems producing a wide range of symptoms [2, 3]. These symptoms include, but are not limited to, cognitive impairment, tremors, ataxia (loss of coordination and muscle movement), hearing loss, pneumonitis, paralysis, shyness and irritability, insomnia, hallucinations, suppressed immunity, renal damage, and other systemic/severe symptoms, including death at lethal doses.

The total amount of mercury released in California spanning the years from 1850–1981 was over 220 million pounds [4]. One gram of mercury, the approximate amount present in old mercury thermometers, is equivalent to an annual atmospheric particulate deposition of mercury in a 20-acre lake and is sufficient to contaminate a lake of that size [5]. In species that have been evaluated, methylmercury (MeHg) has been reported to reduce reproductive success, impair growth and development, alter behavior, and decrease survival.

Teratogens (Greek, “monster-forming”) are environmental agents that cause nonheritable birth defects. Mercury and other heavy metals in the environment act as significant human teratogens; the first large-scale disaster that called public attention to the danger of mercury occurred in Minamata, Japan, in the years after World War II.

In the population surrounding Minamata Bay, a range of severe neurological pathologies that later came to be known as Minamata disease reportedly caused numerous deaths. Methylmercury was found to cause neurological abnormalities observed in nearly 10% of children born near the bay. Symptoms of toxicity, which include staggering, fainting, and loss of muscle control, were found in cats, fish, and birds as well as humans. The cause of these symptoms was traced to mercury contamination of seafood, a dietary food staple for the local population. Methylmercury entering the bay was a by-product of more than 6,000 tons of acetaldehyde produced each year by the Chisso Corporation [6]. This untreated mercury was dumped into the bay, where it contaminated the water and through bioaccumulation entered the food chain to reach the Minamata human population. This tragic example of mercury's harmful effects is directly related to the main focus of this paper, which analyzes sources and effects of mercury bioaccumulation in SFBA and other California ecosystems with their plants, wildlife, and humans.

While we focus in this paper on the SFBA and other locations in California, it is important to note that since the initial studies of Minamata disease, mercury contamination of marine environments has emerged as a problem of global concern. Harmful effects of mercury contamination have been measured in many places around the world. These include the Faroe Islands [7], New Zealand [8], Amazonia [9], Italy [10], Florida [11], Morocco [12], and 17 EU countries [13].

In the Faroe Islands, an initial study showed cognitive deficits in seven-year old children with prenatal exposure to methylmercury [14], a finding supported by follow-up studies carried out 14 years later [15, 16]. Faroe Island marine food constitutes a considerable part of the food source; fish, meat, and blubber from pilot whales are common elements in the diet. The muscle tissue of pilot whales contains both methylmercury and PCBs; pilot whale blubber is especially high in PCBs [17]. Mercury concentrations measured both in maternal hair and in umbilical cord blood exceeded safe levels recommended by WHO [18]. A subsequent study found “Mercury from pilot whale meat adversely affects the fetal development of the nervous system. Decrements in attention, language, verbal memory, and, to a lesser extent, in motor speed and visuospatial function were associated with the mercury exposure. This association was still evident after the exclusion of high exposure subjects” [19].

As with the Minamata case, the highest levels of mercury concentration occur most frequently in coastal or island populations, which often have the greatest access to intertidal and coastal organisms; these residents consume larger amounts of them in their diet. It is important to point out that the effects of mercury toxicity are not limited to coastal or island populations but represent a truly global problem.

## 2. Mercury Speciation and Toxicity

Mercury exists in many forms and oxidation states, both of which play a role in mercury toxicity. The different forms of mercury are highly toxic at relatively low dosages and can produce varying health effects [3, 20]. Toxicity of mercury substances arises mainly from destructive bonding to thiol (-SH), contained in sulfhydryl and selenohydryl groups [21]. In addition, mercury bonds to phosphoryl, carboxyl, amide, and amine groups. Proteins and enzymes with such groups are rendered inactive or inhibited in these reactions with mercury [20], and proper cell functions are inhibited by alteration of protein structure, conformation, and functions [20–22].

The varying forms of mercury can differ in their method of formation, ingestion, and absorption, as well as in their primary targets and level of toxicity. Both in the environment and *in vivo*, mercury interconverts among elemental, inorganic, and organic mercury compounds [1, 20–22].

Mercury crosses the placenta and is considerably more toxic to the fetal brain at approximately one-tenth of the concentration that is toxic to adults. Babies exposed *in utero* and developing children are the most vulnerable and most severely harmed, when exposed to mercury, suffering from possible long-term cognitive impairment, physical maladies, and developmental delays [2, 3, 23]. Commonly used diagnostic modalities to detect mercury levels in humans, such as blood, urine, hair, and fecal analysis, are limited, reflecting only current or recent exposure without assessing total body burden [22].

The three primary forms are elemental mercury (Hg^0^), inorganic mercury (Hg^2+^), and organic mercury compounds (MeHg and EtHg), which are discussed below.

### 2.1. Elemental/Metallic (Hg^0^) Mercury (Zero Oxidation State)

Elemental, or metallic mercury, is unique in being the only metal element that is liquid at room temperature and highly volatile at relatively low temperatures. The main sources of metallic mercury for humans are dental amalgams and workplace exposures [1, 2, 21, 22].

Amalgams are comprised of 50% mercury by weight with the other 50% comprised of other metal alloys. According to the World Health Organization (WHO), human exposure to mercury vapor from amalgam fillings occurs at a rate of 2 to 28 micrograms per filling, per day [22].

Elemental mercury from dental amalgams and other environmental and workplace sources readily vaporizes and is inhaled into the lungs, where up to 80% is absorbed [1, 20–22]. Due to its neutral, monoatomic charge, elemental mercury is lipid soluble and easily diffuses through the alveoli in the lungs and into the bloodstream, where it can be absorbed by numerous tissues. Elemental mercury easily crosses the blood-brain barrier [1, 21, 22] and cell membranes. While the brain and central nervous system are the primary targets of elemental mercury, depositions can also be found in the kidneys, myocardium, skeletal muscles, adrenals, liver, testes, pancreas, and other organs, which contributes to local and systemic dysfunction [1, 21, 22].

### 2.2. Inorganic Mercury (Hg^2+^): Mercuric Ion (2+ Oxidation State)

Once inside cells, elemental mercury (Hg^0^) is oxidized to form the highly reactive, mercuric ion (Hg^2+^). The mercuric ion inhibits or interferes with a host of enzymes and with a number of metabolic processes necessary for homeostatic cellular function [1, 2, 21]. The mercuric ion is less mobile, and because charged particles do not cross membranes easily, it is less able to exit the cell due to its positive charge [1, 2]. Because of this, once oxidized in the brain, mercury can remain trapped in cells, maintaining detectable levels many years after initial exposure [1, 2, 20]. Organisms, including humans, depend on selenium for certain functions carried out throughout the body by selenoproteins.

The body depends on selenium and selenoenzymes for antioxidative protection. The mercuric ion has an extremely high affinity for selenium and selenoproteins, which are vital to cellular redox regulation [1, 21]. The bonding of selenium to Hg^2+^ becomes nearly impossible to reverse. Based on autopsy data of humans with proven mercury poisoning, this bonding plays a key role in its long-term retention in the brain which is estimated to be 17+ years [1]. In comparison, the half-life of mercury in the body in general is an estimated 60 days [1, 20, 22]. At extremely low levels, 3.6 parts per trillion (ppt), Hg^2+^ decreases glutathione, a vital antioxidant, and increases oxidative stress that can lead to cellular toxicity and apoptosis [1, 21].

Animal and *in vitro* studies have demonstrated that inorganic Hg^2+^ replicates all physical and pathological symptoms observed in Alzheimer's patients. These changes include production of inflammatory amyloid plaques, production of reactive oxygen species (ROS), and hyperphosphorylation of the *Tau* protein causing neurofibrillary tangles [1]. The apolipoprotein E (ApoE) transporter protein genotype is an identified genetic risk factor for Alzheimer's disease. The ApoE4 genotype relays a 15-fold risk relative to the ApoE3 and the ApoE2 genotypes, which appear to have protective qualities [1].

Mercury exposure at toxic levels most commonly affects neurologic, renal, and gastrointestinal systems producing a wide range of symptoms [2, 3]. These symptoms can include cognitive impairment, tremors, ataxia (loss of coordination and muscle movement), hearing loss, pneumonitis, paralysis, shyness and irritability, insomnia, hallucinations, suppressed immunity, renal damage, and other systemic/severe symptoms, including death at lethal doses. Mercury crosses the placenta and is considerably more toxic to the fetal brain at approximately one-tenth of the concentration that is toxic to adults. Babies exposed *in utero* and developing children are the most vulnerable and most severely harmed, when exposed to mercury, suffering from possible long-term cognitive impairment, physical maladies, and developmental delays [2, 3, 23].

Commonly used diagnostic modalities to detect mercury levels in humans, such as blood, urine, hair, and fecal analysis, are limited, reflecting only current or recent exposure without assessing total body burden [22].

### 2.3. Organic Mercury: Methylmercury (MeHg) and Ethylmercury (EtHg)

Mercury, in its elemental and inorganic states, is routinely altered to its organic form, methylmercury, through the metabolic processes of microorganisms, particularly some iron- and sulfate-reducing bacteria [24]. Microbes and abiotic processes, along with pH (lower pH favors methylation) and dissolved organic carbon (DOC) levels in water, control mercury methylation in an aquatic environment [25, 26]. In an aquatic environment, the highly reactive Hg^2+^ is the base for methylation, creation of MeHg, and other organic mercury species [25]. Of particular concern is the known bioaccumulation, concentration, and magnification of methylmercury in aquatic food webs [3, 25, 27].

Progressively elevated levels of mercury in food chains can reach levels exponentially higher in the top predators, as compared to mercury levels in surrounding waters. The major source of MeHg for humans is contaminated seafood [3, 22, 26, 27].

Americans consume on average about 2.4 *μ*g of mercury per week through fish consumption, 2.3 *μ*g of which is absorbed [1]. National and global agencies have set guidelines, which often do not indicate total mercury loads, for mercury exposure. The World Health Organization (WHO) and Environmental Protection Agency (EPA) established the following mercury guidelines:

- World Health Organization (WHO)-Mercury Standard Guidelines [18]: WHO-human-methylmercury (0.23 *μ*g/kg/day)
- Environmental Protection Agency (EPA)-Mercury Guidelines [18]: EPA-human-methylmercury (0.1 *μ*g/kg/day)

The maximum allowable daily mercury intake from the two government sources differ significantly, illustrating the difficulty of establishing such guidelines on known highly toxic substances within mass populations. The relatively large discrepancy is mainly attributable to hypothetical inferences taken from a variety of sources, none of which are drawn from clinical human trials performed with proper protocols. However, these extremely low WHO and EPA numbers, at 0.23 *μ*g/Kg/day and 0.1 *μ*g/Kg/day, illustrate the perceived high level of toxicity of mercury by both agencies.

### 2.4. Synergism Amplifies Mercury Toxicity

Synergistic toxicity refers to a combined toxicity of two or more poisonous chemicals that is considerably greater than the toxic effect of the chemicals taken separately. Data are available on the individual toxicities of heavy metals such as mercury, lead, cadmium, and an array of other elements and compounds (e.g., arsenic, PCBs, BPA, and organophosphate pesticides (glyphosate in formulations such as Roundup). Determining the combined toxicity of multiple toxins may be equally, if not more, important than quantifying harmful effects of single toxins. For example, in a much-cited study by Shubert et al. [28], the relationship between mercury and lead was observed to be synergistic. The experiment used the lethal dose of both mercury and lead salt that would each kill 1/100 (LD_1_) mice. The combined salts were then administered to mice, killing 100/100 (LD_100_) of them. This level of increase in lethal toxicity strongly suggests that the combined salts had a multiplier effect, causing a much higher LD than what would have been predicted for a mere combination of the mercury and lead salts.

It is difficult to test precisely for the synergistic interactions among heavy metals due to their complex relationships in actual biological systems; “each metal may be involved in a spectrum of metabolic pathways to elicit specific toxic effects.” Yet in one study of synergistic toxicity of multiple heavy metals which started using a lethality test using a single metal and then followed it with lethality tests using multiple metals, the results showed that even minute amounts of each metal in concentration could generate a severe lethal impact on *Caenorhabditis elegans*, a free-living nematode species [29].

The widespread use of pesticides in public health and agriculture is responsible for both environmental pollution and health hazards. “A widespread and repeated exposure of both human and animal populations to mercury combined with other heavy metals and pesticides has been observed. Heavy metals and pesticides in combination may have a more severe impact on health than their individual effects” [30]. Despite its general high standard of living, the United States ranks 46^th^ in infant mortality rate (IMR) among nations in the report submitted to the World Health Organization. In addition to giving us key information about maternal and infant health, IMR is a well-known marker of a society's overall health.

Environmental toxicants may be one factor responsible for increased infant mortality. When mercury, aluminum, and lead interact synergistically with fluoride compounds, they produce metal fluoride complexes which can disrupt the signaling processes that control and guide development. Pesticides may play an important role: “Among the interactants are glyphosate and phosphate containing fertilizers that end up in the food and water because of their widespread use in agriculture” [31].

In developing countries, particularly in sub-Saharan Africa, “increased artisanal mining activities, illegal refining, use of leaded petrol, airborne dust, arbitrary discarding of toxic waste, absorption of production activities in inhabited areas, inadequate environmental legislation, and weak implementation of policies” have all added to the public health burden caused by heavy metal mixtures (including mercury) [32]. These preliminary studies highlight the importance of further research in determining which other known toxic substances have synergistic effects with mercury and what the nature of these synergistic effects is. Pollutants in the environment are rarely ever present singly, occurring instead in mixtures with largely unknown toxicity potentials.

### 2.5. Global Sources of Mercury Pollution

**Figure 1:**
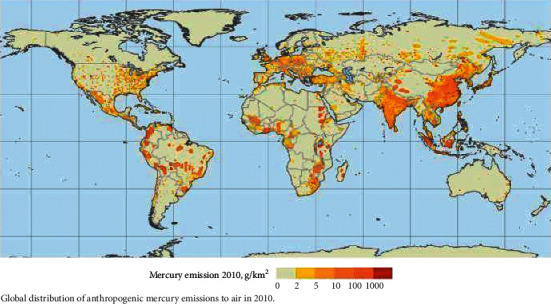
Global distribution of anthropogenic mercury emissions to air in 2010. Figure source: United Nations Environment Program [26].

Natural sources of mercury pollution include volcanic activity, forest fires, and soil emissions. Although mercury compounds have multiple applications, including those in industry and dentistry, an increasing understanding of mercury's toxicity has led to progressive restrictions in its use [3, 21]. Global contributions of mercury pollution in 2010 are illustrated in Figure 1. Asia accounts for the greatest proportion of anthropogenic mercury emissions, contributing over 50% of the total global distribution.

**Figure 2:**
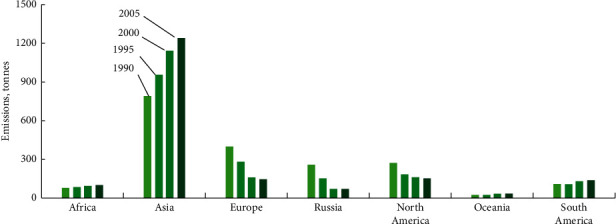
Anthropogenic global mercury emissions, tons, 1995–2005. Figure source: United Nations Environment Program [26].

**Figure 3:**
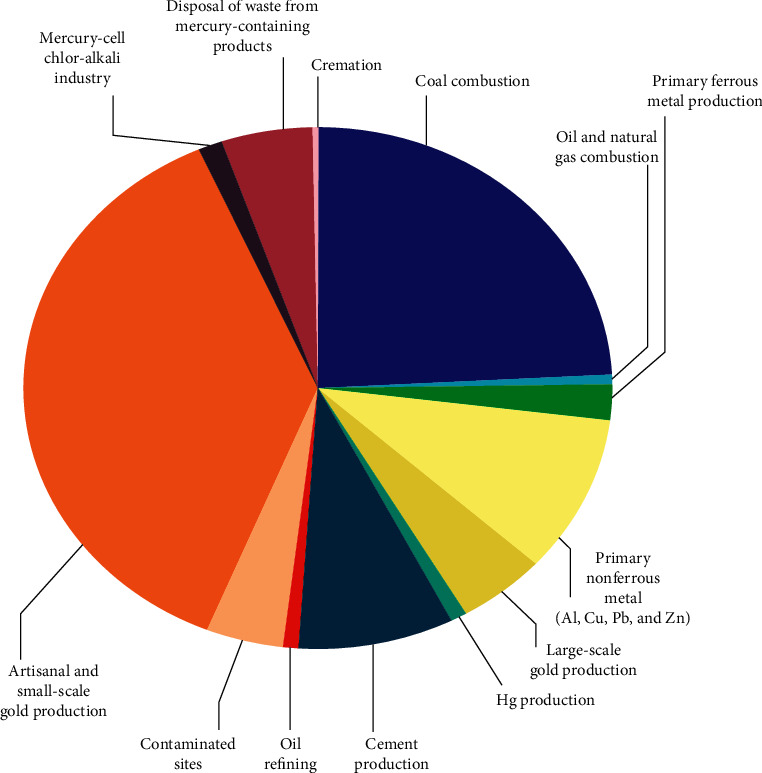
Data for 2010 mercury emissions from the highest emitting industry sectors, from the 2013 UNEP Global Mercury Assessment. Figure source: United Nations Environment Program [26].

Global anthropogenic sources of mercury pollution in the environment include coal-burning plants, chlorine factories, and gold mining/processing activities (see Figures 2 and 3). As indicated in Figure 2, China emits three quarters of the East and Southeast Asian emissions and is solely responsible for approximately one-third of the total global pollution load [26]. Other regions emit mercury, decreasing in the following order: Europe, North America, South America, Russia, and sub-Saharan Africa. As clearly illustrated in Figure 2, Asia was the only region that significantly increased its mercury output between 1995 and 2005.

The EPA [33] quantifies approximate anthropogenic industrial contributions of mercury to the air (see Figure 3). Annual contributions include artisanal and small-scale gold mining (37%, 727 tons), fossil fuel burning (25%, (484 tons), nonferrous metal production (10%, 193 tons), cement production (9%, 173 tons), large-scale gold production (5%, 97 tons), waste disposal (5%, 95 tons), contaminated sites (4%, 82.5 tons), primary ferrous metal production 2%, 45.5 tons), and the chlor-alkali industry and crematoriums (1%, 4–6 tons each).

### 2.6. Sources of Mercury in the San Francisco Bay Area (SFBA)

Miners used mercury, also known as quicksilver, to recover and purify gold from ore throughout the Western United States. Mercury was used in the process of amalgamation (physical combination) because of its relatively low boiling point for a metal (357°C), which is less than that of gold (2,970°C). Mercury also has a high density, which allows gold-mercury amalgams to sink and settle. Most of the mercury used in gold recovery was obtained from mercury deposits in the Coast Range on the West side of the Central Valley [34].

After the Gold Rush, notable amounts of mercury-contaminated sediments remained at mining sites, especially in drainage tunnels. Sources of mercury contamination in the SFBA include contributions from mercury mines in New Almaden, which lies adjacent to the Guadalupe River watershed and drains into South San Francisco Bay. In addition, gold mining in upstream portions of the Sierra Nevada Mountains played a significant role in adding mercury to the SFBA environment and in other local watersheds [35].

**Figure 4:**
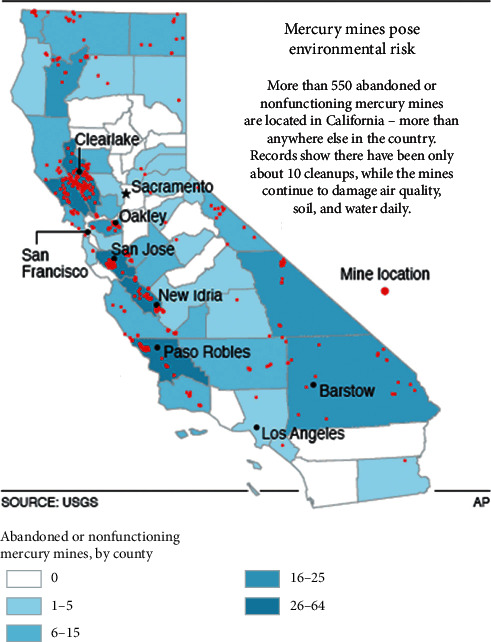
Map of mercury mines in California. Figure source: United States Geological Survey [35].

**Figure 5:**
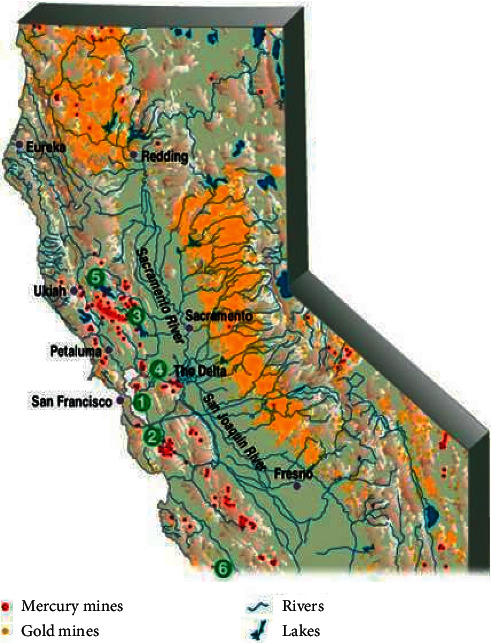
Map of gold mines and mercury waterways in California.

In 2000, the United States Geological Survey [35] reported that some 550 abandoned and inactive mercury mines in California continued to cause environmental damage (see Figure 4). The map in Figure 5 highlights sites of mercury *(red)* and gold mines *(yellow)* throughout northern and central California and their connective water tributaries. Figure 5 shows the major pathways through which mercury contamination can occur in sediment and water.

Placer mining in California released close to 10 million lbs of mercury to the environment. About 80–90% of it was concentrated in the Sierra Nevada Mountains [36]. Three million lbs of mercury were lost in hardrock mines, which crushed gold ore using stamp mills. Mercury production in California, spanning the years 1850–1981, totaled well over 220 million lbs, and it peaked in the 1870s [36, 37]. For instance, a gold pan could be found with over 30 g of mercury from just 1 kg of mercury-contaminated sediments in a drainage tunnel.

### 2.7. Environmental Bioaccumulation of Mercury: Movement from Air, Water, and Sediments into Biological Systems

Landers et al [38] assessed fate, transport, and ecological impacts of mercury and other airborne contaminants through a study in National Parks of Western USA. They report mercury can be carried to these parks, both on fine particulate matter and as gaseous compounds. Gaseous elemental mercury (Hg^0^) has a lifetime up to one year or more, which results in a fairly uniform global reservoir of elemental mercury in the atmosphere [38]. Elemental mercury slowly oxidizes to Hg^2+^, which is then quickly removed via wet or dry deposition. Similarly, Wright et al [39] investigated mercury deposition at the coast of California. Mercury is present in the atmosphere as elemental gaseous oxidized compounds. In air (Table 1), mercury is generally found in low concentrations (parts per trillion (ppt)). However, aerial mercury concentrations at industrial (e.g., coal burning power plants and oil refineries) sites occur at higher concentrations. Mercury is broadly distributed throughout much of the SFBA, with most of it from aerial transport, historic mining, and weathering of cinnabar, a natural mercury ore. The North Bay receives over 95% of the total freshwater inflow, while the South Bay receives less than 5%.

Long and Morgan [42] reported in 1990 on sediment-sorbed contaminants as part of the National Oceanic and Atmospheric Administration's “national status and trends program.” Their extensive summary evaluated biological effects of mercury and other toxins. It included a review of published information on mercury concentrations in *estuarine* and coastal marine sediments. Only a moderate amount of sediment data were available for the US but did include two studies in San Francisco Bay. One study reported 2.3 ppm mercury dry weight (Table 1).

As might be expected, mercury is also found in terrestrial soils. An Environmental Protection Agency (EPA) report [43] reviews and summarizes data on mercury content of virgin and cultivated surface soils from several countries; average concentrations ranged from 20 to 625 ng/g (0.020 to 0.625 ppm). The EPA review explains the highest concentrations were generally found in soils from urban locations and in organic rather than mineral soils. Content of most soils varies with depth, with the highest mercury concentrations generally found in surface layers.

**Figure 6:**
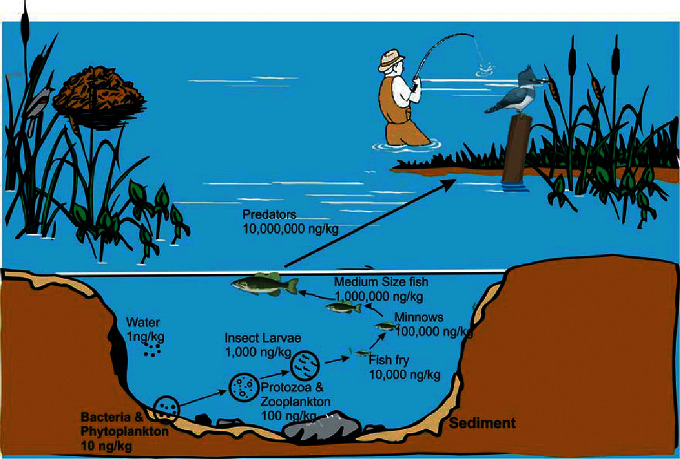
Mercury biomagnification in an aquatic and riparian food chain. Source: New Jersey Department of Environmental Protection, Mercury Task Force [44].

Figure 6 conceptually illustrates biomagnification of mercury concentrations up through food webs in aquatic ecosystems, starting with bacteria converting inorganic mercury to methylmercury. Upon methylation, mercury enters food chains, where organisms successively bioaccumulate mercury up to 10 times greater concentrations than the level in their food source. To illustrate this example of increasing magnitude, bacteria and phytoplankton have a mercury concentration of 10 ng/kg (10 ppt), followed by zooplankton and protozoa with a concentration of 100 ng/kg (100 ppt). The level of concentration continues to increase up through the trophic levels of this food chain until the apex predator, the kingfisher (in this example), is reached. Apex predators, such as the kingfisher, can bioaccumulate concentrations up to several million times greater than the concentration in water.

Biomagnification processes may be compounded by water acidification. Substances including sulfur, which provide reactants required for methylation, result in conversion of mercury to organic mercury. Much remains to be understood about some processes in the biogeochemical cycle of mercury, including its methylation (creating MeHg, the bioavailable form of mercury), subsequent bioaccumulation in biota, and resulting biomagnification in the food web.

## 3. Mercury Concentrations up through Trophic Levels

Species are often classified into five trophic levels, which are based on the transfer of energy at each level, i.e., whether they synthesize or consume energy. Some organisms create their own energy, while others receive energy by feeding on other organisms, typically lower in the food chain. In this report section, we provide available examples of SFBA organisms reported to contain mercury at each trophic level. However, as some information gaps exist, the text and tables are supplemented with data and discussion of mercury-laden species from other locations in USA and other countries.

### 3.1. Trophic Level I: Autotrophic Bacteria, Algae, and Plants That Produce Their Own Energy (Autotrophic Primary Producers)

Examples: phytoplankton, diatoms, and kelp.

Most methylmercury taken up by aquatic biota comes from microbes that process inorganic, divalent mercury, Hg^2+^, in anaerobic sediments facilitated by some sulfate- and iron-reducing bacteria [45]. Sulfate-reducing bacteria and archaea convert sulfate (SO_4_^2^-) into hydrogen sulfide (H_2_S), essentially anaerobically “breathing” sulfate rather than oxygen. Methylmercury production relies on availability of inorganic mercury (Hg^2+^) for assimilation and methylation. The capacity for methylation of mercury depends on the strain (genetic variant or subtype) of algae or bacteria [45]. For example, in sulfate-reducing bacteria, methylation can occur through passive diffusion or an active transport mechanism [45]. The methylated mercury that does not accumulate in the bacteria is excreted as methylmercury and becomes available for uptake by diatoms, desmids, and aquatic rooted plants. All of these organisms, which are at the base of the food chain, are then consumed by organisms from higher trophic levels.

The phenomenon of methylation takes place as Hg^2+^ ions segregate, primarily in the outer membranes of cells. Methylmercury passes into the cytoplasm of a cell where assimilation efficiency is greater. This crucial step of methylation of inorganic Hg^2+^ to organic, MeHg, which is fundamental to the entry of mercury into the food chain and its further biomagnification at higher trophic levels, is not fully understood.

Algae also play a vital role in trophic transfer of MeHg to fish. The greatest bioconcentration (i.e., uptake from water) takes place in these plants, which essentially function as “mercury sponges” [46]. Methylmercury assimilation by algae begins with an adsorption interaction at the aqueous solution-cell membrane interface. The toxin is then transported across the membrane barrier. Reactions take place within the cytoplasm and nucleus of the cell [46]. As with bacteria, details of binding mechanisms and assimilation are unknown.

Kuwabara and coinvestigators [47] provided an example of MeHg concentrations in phytoplankton from Lake Almaden, located in the SFBO Area several miles south of San Francisco Bay. Their collections for analysis consisted primarily of the taxa *Merismopedia glauca*, *Cryptomonas erosa*, and *Aphanothece smithii*. MeHg content ranged from less than 1.50 (the low detection limit) to 8.2 nanograms per gram dry weight (Table 2). These concentrations in phytoplankton were higher than those of the Kuwabara team found in Guadalupe Reservoir, which is also located just south of the SFO Bay Area. Methylmercury in this reservoir's phytoplankton was undetectable.

### 3.2. Trophic Level II: Plant Consumers (Primary Consumers)

Examples: clams, cod, and sardines.

Trophic level II organisms, generally herbivores and small-prey fish, typically consume autotrophic level I organisms, such as rooted plants and algae. Herbivores, such as some zooplanktonic species, feed on phytoplankton. Trophic level II species often have MeHg concentrations of a few ppb. For example, investigators have reported concentrations of 0.009 ppm (9 ppb) in zooplankton and filter-feeding clams. However, for some primary consumers, concentrations of MeHg can reach nearly 5 ppm, as shown in Table 3.

Thus, MeHg is introduced to other higher-level (trophic level II–V) organisms. Methylmercury does not dissolve or break down; it binds to proteins found in visceral, muscular, and adipose tissue. Fish assimilate MeHg rapidly but excrete it slowly due to its insoluble nature. This higher rate of MeHg assimilation versus its slow excretion results in its progressive trophic level accumulation. In fish, 95% of MeHg is readily absorbed in the gastrointestinal tract. Because of this accumulation, fish are one of the most significant sources of consumption-related MeHg exposure to humans and other animals.

Acute MeHg contamination in fish generally results in inflammation of gill covers, increased respiratory activity, loss of homeostatic function, lethargy, and death [50]. Sublethal or chronic exposures to MeHg can manifest as growth inhibition, behavioral anomalies, metabolic disturbances, reproductive failure, changes in blood chemistry, osmoregulatory complications, and altered gas exchange in both marine and freshwater organisms [51].

### 3.3. Trophic Level III: Carnivores That Consume Herbivores (Secondary Consumers)

Examples: dragonfly larvae, California clapper rail, California least tern, and perch.

Level III organisms are found in various taxonomic groups of invertebrates and vertebrates. These animals have higher methylmercury levels than found in trophic levels I and II organisms. Examples of level III organisms include some groups of insects, fish, and birds (see Table 4). Birds vary greatly in the amount of MeHg in their bodies, depending on factors such as diet and feeding grounds. Fruit- or seed-eating birds in trophic level II have much smaller concentrations of MeHg than do piscivorous (fish-eating) and other carnivorous birds in trophic levels III and higher. While birds feeding on grains generally have low concentrations of MeHg in their bodies, grains treated with mercury-containing fungicides can cause either acute or chronic effects in these birds [54].

In avian species, areas of mercury concentration are feathers, liver, other internal organs, and eggs. Sampling feathers is advantageous for several reasons: it is nondestructive, does not require refrigeration for storage, and can be easily compared with feathers archived decades ago. Mercury has a high affinity for sulfhydryl groups present in proteins, and developing feathers are comprised of keratin, a sulfhydryl-rich protein.

**Figure 7:**
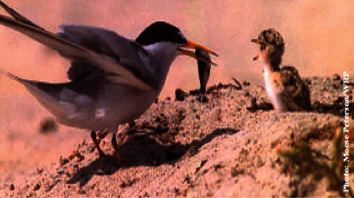
California least tern in trophic level III is a small endangered seabird exhibiting pathologies related to MeHg contamination. It typically inhabits lagoons or shallow estuaries, where it feeds on the abundant small fish. Photo source: California Dept. of Pesticide Regulation (cdpr.ca.gov). Photo by Moose Peterson of Wildlife Photography, http://www.moosepeterson.com.

The California least tern, *Sterna antillarum browni* (Figure 7), and the California clapper rail, *Rallus longirostris obsoletus*, both Federally-listed as endangered, also exhibit pathologies related to MeHg contamination. In tidal marshes of the SFBA, recovery of the California clapper rail may be in peril due to MeHg toxicity. In a study by Heinz et al. [55] methylmercury was injected into eggs. The hatchability in clapper rails was found to be adversely affected by MeHg. Bioaccumulation of MeHg in SFBA ecosystems is specific to species demographics, due to variation of MeHg concentrations found in different habitats. Eggs of piscivorous birds collected from San Pablo Bay and Suisun Bay (northern part of the SFBA) had concentrations ranging from 0.28–0.70 ppm, while eggs from the South Bay (southern part of the SFBA) ranged slightly higher, i.e., from 0.56–1.05 ppm.

**Figure 8:**
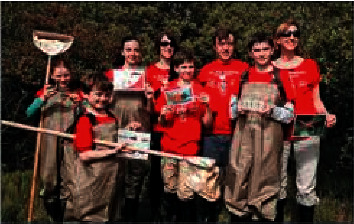
A team of scientists and citizens (young students in this case) in 2015 collecting dragonfly larvae at Golden Gate National Recreation Area in San Francisco. Photo source: United States Geological Survey [4].

The National Park Service is monitoring MeHg distribution in National Parks throughout the country by examining levels of MeHg present in various species of dragonfly larvae, which serve as important sentinel organisms. This monitoring effort is conducted by scientists along with citizens [56] in 22 national parks and one state (GA) park across the US, including Golden Gate National Recreation Area (“GOGA”) in San Francisco. This project has an educational component in which students from different age groups (see Figure 8) are trained to follow protocols for collecting dragonfly larvae). Specimens from dragonfly larvae collected in selected freshwater marshes of GOGA had a mean concentration of 0.343 ppm [52], which is within the order of magnitude of concentrations expected for organisms in trophic level III. Of dragonfly larvae evaluated in the 23 sites mentioned, those from GOGA had the highest mean concentration of total mercury. Concentrations at the 23 sites ranged from below detection limit to 0.844 ppm dry weight, and the overall median concentration for all sites was 0.112 ppm.

### 3.4. Trophic Level IV: Carnivores That Consume Other Carnivores (Tertiary Consumers)

Examples: common loon, Forster's Tern, jacksmelt, and Chinook salmon.

Organisms in trophic level IV are tertiary consumers, and they have greater MeHg accumulations than animals in lower trophic levels. Among level IV animals are some species of migrating birds that pass through the SFBA. Wetlands in this area are integral to survival and reproduction of large bird populations, and many avian species are sensitive to methylmercury, particularly during developmental stages as embryos and chicks [55]. Consequently, they face reproductive complications.

**Figure 9:**
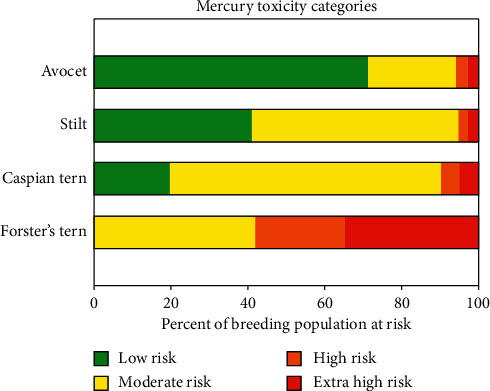
SFBA native bird species in trophic level IV are at varying risks from mercury toxicity. This figure shows relationship between MeHg levels and diet. Those at higher trophic levels, e.g., Forster's tern, have a greater risk due to their consumption of more highly contaminated mercury-tainted organisms. Figure source: Ackerman et al. [57].

The American avocet *(Recurvirostra americana)*, Forster's tern *(Sterna forsteri)*, Caspian tern (*S*. *caspia*), and black-necked stilt *(Himantopus mexicanus)* all inhabit areas in and around managed ponds in SFBA (see Figure 9). Ackerman et al. [57] have reported about 48% of breeding Forster's terns and nearly 5% of stilts, avocets, and Caspian terns have a high MeHg blood concentration, i.e., >3.0 ppm (see Figure 9). At this concentration, common loons (*Gavia immer*) experienced a 40% decrease in reproductive success. The annual mean in MeHg concentrations in Forster's tern eggs varied from 0.9–1.6 ppm in 2009. In the South Bay, downstream of the New Almaden Mining Site, concentrations of MeHg found in both eggs and blood samples of this species have been consistently higher.

Trophic level IV species, in summary, tend to feed on other carnivores that bear moderate or high MeHg loads. As a result, the former species incorporate and further concentrate the toxin to their detriment. For example, halibut reported with a MeHg level of 0.241 ppm (Table 5) were likely to experience reproductive complications [49].

### 3.5. Trophic Level V: Apex Predators (Final Consumers)

Examples: sharks, eagles, bears, and humans.

At trophic level V are apex predators, animals tending to have a long life span and larger body size. Because they consume prey from lower trophic levels (i.e., III or IV), apex predators have the highest “body burdens” of MeHg. Humans are no exception and can be exposed to high concentrations of MeHg from eating seafood.

The gray smooth-hound shark (*Mustelus californicus*) is found in California, including the San Francisco Bay estuary. It feeds on mollusks, clams, crabs, shrimp, small fish, and squid. Although these food items have lower concentrations of MeHg, this shark consumes them in high amounts. Largemouth bass (*Micropterus salmoides*) are native to North America, including California and the SFBA. This ubiquitous game-fish species is commonly found in reservoirs and lakes. As such, this bass provides a sound baseline of MeHg concentrations for apex species in aquatic habitats and can be used for fish consumption advisories [58]. Largemouth bass, which occur in some reservoirs in the Guadalupe River watershed (New Almaden Mining District), have among the highest MeHg concentrations (e.g., 6.6 ppm) observed in the entire United States [53].

A 1994 survey of fish in the SFBA found levels of mercury ranging from 0.07–1.26 ppm in species popular with human consumers. High concentrations were found in the leopard shark (*Triakis semifasciata)*, brown smooth-hound shark (*Mustelus henlei*), white sturgeon (*Acipenser transmontanus*), and striped bass (*Morone saxatilis*). Striped bass from the SFBA have the highest average concentration with a mean of 0.44 ppm in fish 60 cm long [53]. Fish with lower mercury levels include Chinook salmon (*Oncorhynchus tshawytscha*), jacksmelt (*Atherinopsis californiensis*), brown rockfish (*Sebastes auriculatus*), and red rock crab (*Cancer productus*). Fish with higher mercury levels tend to be large, apex predators in both fresh and marine systems. Among these are walleye (*Sander vitreus*), striped bass, swordfish (*Xiphias gladius)*, yellow tilefish (*Hoplolatilus luteus)*, king mackerel (*Scomberomorus cavalla)*, and brown smooth-hound shark. The mercury concentration of 3.5 ppm in this shark is among the highest levels detected in apex predators.  Table 6 summarizes concentration levels of mercury in selected trophic level 5 organisms. No consistent reliable data exists on general levels of mercury concentration in humans due to variable diets and other environmental factors.

## 4. Sources and Measurement of Mercury Contamination in Humans

Mercury is uniquely harmful when released into the environment, due to its toxicity and its long-term persistence in ecosystems through recycling. Mercury is mobile when incorporated into organic compounds, principally in the form of methylmercury. It accumulates and concentrates dramatically as it is biomagnified through terrestrial and marine food webs. Global anthropogenic mercury emissions pose immediate and long-term challenges for many species, including humans. Mercury is “the only metal representing a volatile gas at room temperature, which is readily absorbed (80%) by the respiratory system” [1].

Data and evidence summarized from studies cited in this paper demonstrate how and to what extent mercury moves through the environment and food chains [38].

Air transports mercury worldwide and releases it in the form of precipitation, contaminating water bodies, groundwater, sediments, and subsequently ecosystems. Measured concentrations of mercury yield convincing evidence of biological amplification [47].

Concentrations magnify exponentially, starting with primary producers of the first trophic level, including bacteria and algae, and reaching toxic levels in apex species, such as sharks, killer whales, and polar bears. Humans fall into the upper end of trophic levels and are among those species with the highest potential to capture mercury and concentrate it in organs and tissues.

In addition to sources of mercury already discussed, humans are potentially exposed to mercury from such sources as dental amalgams and vaccines. Amalgams, commonly referred to as “silver fillings,” continue to be used in dentistry to fill cavities from tooth decay. Amalgams are alloys of liquid mercury mixed with powdered metals consisting of tin, copper, and silver. Elemental mercury comprises about 50% of these amalgams by weight [60]. Mercury is the only metal in a liquid state at room temperature, allowing it to bind well with the powdered alloy [61].

These filling materials are cost-effective and durable, and for this reason mercury amalgams continue to be used but not as widely as they once were. Many dentists now choose to use alternative materials such as resin and composites for health reasons. Although several countries in Europe, including Norway, Sweden, and Denmark, have banned the use of mercury in dental amalgams, mercury is still being used in the US for dental fillings [31].

Small amounts of mercury vapor are released during mechanical processes such as chewing and are absorbed through the lungs by inhalation and ingestion. Concentrations of mercury can increase through bruxism or teeth grinding. Other factors, such as oral temperature, pH, and surface area of the amalgam itself, are contributing factors to mercury concentration. Exposure to high levels of mercury vapor have been linked to adverse effects in the kidneys and brain. Susceptibility to this neurotoxin varies among individuals, depending on their genotype [62]. Studies such as the Children's Amalgam Trials suggest that exposure through dental amalgams can be deleterious to those who possess susceptible genes. In addition, there is evidence that the use of mercury containing amalgams may be affecting dentists, dental hygienists, and their assistants as well as patients: “Dental workers have higher levels of mercury as measured in blood, urine, stool, nails, hair, and organs” [61].

Current diagnostic modalities available to determine mercury in humans include urine, blood, and hair analysis. These methods typically show recent, acute, or chronic exposure to mercury, but do not measure deposited mercury levels in tissues. Mercury is measured in various tissues and organs of nonhuman organisms, but almost exclusively in urine, blood, and hair of humans. In addition, total mercury load in humans up to this point cannot be determined exclusively by one test; urine analysis yields elemental mercury load, while hair and blood analyses identify organic mercury loads.

The meta-analysis study by Bernhoft, Mercury Toxicity and Treatment, a Review of Literature, found the following: “In addition to the brain, metallic mercury is also deposited in the thyroid, breast, myocardium, muscles, adrenals, liver, kidneys, skin, sweat glands, pancreas, enterocytes, lungs, salivary glands, testes, and prostate and may be associated with dysfunction of those organs. Mercury also has affinity for binding sites on the surface of T cells and for sulfhydryl groups influencing T cell function. Mercury deposits readily in placenta and fetal tissues and is found in breast milk” [22].

Knowing human health is challenged by both acute, chronic exposure and accumulation of mercury from food and environmental sources, it is extremely important to develop more sensitive methods of measuring total mercury load. One possible promising development is the patented liquid chromatographic mercury speciation technology used by Dr. Christopher Shade to create the Mercury Tri-Test, which measures urine, blood, and hair levels [63]. Early detection of total mercury load would help to prevent human pathologies associated with mercury exposure from developing over time. These maladies include kidney, heart disease, and cognitive degeneration.

As discussed earlier, large amounts of mercury were dumped into Minamata Bay, Japan, starting in the 1930s, resulting in a population devastated by harmful effects of mercury contamination. One of the major achievements of the international community has been the Minamata Convention on Mercury, an international treaty designed to protect human health and the environment from anthropogenic releases of mercury and its secondary compounds.

## 5. Recommendations to Minimize Human Mercury Exposure

In October, 2013, more than 90 countries signed the Minamata Convention on Mercury, an initiative begun in 2009 by the Governing Council of the United Nations Environment Program (UNEP) (http://www.mercuryconvention.org/Portals/11/documents/conventionText/Minamata%20Convention%20on%20Mercurye.pdf).

The Minamata Convention on Mercury is a global treaty to protect human health and the environment from the adverse effects of mercury. The convention is designed to recall the name of the Japanese city that went through the devastating episode of mercury poisoning that first brought the extreme dangers of mercury to global awareness. The Minamata treaty presented comprehensive recommended measures to reduce mercury emissions globally. The goal of this pact is to “…protect the human health and the environment from anthropogenic emissions and releases of mercury and mercury compounds.” The participating members in the treaty are expected to reduce their mercury emissions by complying with key provisions designed to curb both intended and unintended future emissions of mercury and its use by leading contributing industries.

Among its requirements are cutting edge emission‐control technologies on new coal‐fired power plants, boilers, and smelters; in addition, it bans the use of mercury in the production of acetaldehyde. Certain controversial uses of mercury were exempted, including artisanal gold mining, the use of dental amalgams, and as a preservative in vaccines. Further reductions in mercury usage and emission must be brought about by public education, combined with strong regulations effectively enforced through legal systems to ensure compliance. Only such comprehensive efforts will bring about desired long-term reductions in levels of mercury in the environment.

The primary exposure to methylmercury occurs through the consumption of contaminated seafood [64]; in some inland areas it is through rice grown in contaminated waters [65]. Methylmercury in the diet is clearly toxic for the human brain, kidney, liver, heart, and nervous system; it is particularly dangerous for the developing fetus, as earlier cited work shows (e.g., Minamata and Faroe Islands). Mercury exposure during pregnancy can cause lasting deficits in development of a child's brain and nervous system. In 2001, the EPA concluded that a pregnant woman could consume 0.1 micrograms of mercury per kilogram of bodyweight daily without ill effects to her fetus and that this amount of mercury would also be safe for children and adults [66]. Since then, however, some studies have found measurable damage to infants' brain development in mothers exposed to lower levels of mercury. Several scientists and advocates specializing in mercury damage have concluded that the EPA's safe level is too lax to protect the developing fetus, and recommendations have been made that the EPA lower its mercury exposure level by 50 to 75% [67, 68].

While general fish advisories can be found at the EPA website (https://www.epa.gov/mercury/guidelines-eating-fish-contain-mercury and both state and regional advisory ones as well (https://fishadvisoryonline.epa.gov/AdvisoryDetails.aspx?ADVNUM=27), these government websites are not easily accessible or user-friendly. The Monterey Bay Aquarium is a neighbor of SFBA, with its own fish advisory (https://www.seafoodwatch.org/-/m/sfw/pdf/whats%20new/complete%20recommendation%20list.pdf.

In our judgment, the Environmental Working Group (https://www.ewg.org/research/ewgs-good-seafood-guide publishes the clearest and most user-friendly guide to wise fish consumption. The Environmental Working Group's (EWG) extensive analysis of the latest scientific research on seafood points consumers to fish lowest in mercury contamination, highest in omega-3 fatty acids, and most sustainably produced (https://americanpregnancy.org/pregnancy-health/omega-3-fish-oil-and-pregnancy/).

The EWG website contains a Seafood Calculator for making a custom seafood list based on age, weight, gender, and other personal information (https://www.ewg.org/research/ewgs-good-seafood-guide.

- It includes a well-documented Good Seafood Guide which most highly recommends wild salmon, sardines, mussels, rainbow trout, and Atlantic mackerel: one or two four-ounce servings a week of these fish have little mercury and optimum levels of omega-3 fatty acids for pregnant or nursing women and people with heart disease.
- Oysters, anchovies, pollock, and herring are also healthy choices: these species have favorable concentrations of omega-3 fats. One four-ounce serving provides at least 25 percent of the weekly recommended omega-3 consumption. A pregnant woman of average weight could eat three four-ounce servings per week without ingesting too much mercury.
- Shrimp, catfish, tilapia, clams, and scallops are low mercury but also low omega-3 sources. They can be healthy sources of protein and other nutrients, but an adult would have to eat five to 20 four-ounce portions to meet the omega-3 recommendation for pregnant women and people with heart disease.
- Canned light and albacore tuna, halibut, lobster, mahi-mahi, and sea bass contain too much mercury to be part of the regular diet of pregnant women and children; the safe amount in the diet depends on age, weight, and health status.
- The following fish are to be avoided on account of high mercury content: shark, swordfish, tilefish, king mackerel, marlin, bluefin and bigeye tuna, and orange roughy. This high mercury seafood should never be eaten by pregnant women and children, and it should be eaten by others only infrequently or not at all.

A historical factor needs to be mentioned. The SFBA generally has higher levels of mercury in its ecosystems than other urbanized regions of mainland United States, largely due to the problematic legacy of California's history of gold mining. Local environmental measures to decrease mercury emissions would include more research to curb erosion and mercury movement into the environment from the more than 550 abandoned gold mines in California. Unfortunately, no data exists regarding local human health issues resulting from the higher levels of mercury. Mercury pervasiveness, combined with long-term human exposure, may well play a contributing role in the etiology of chronic long-term, degenerative conditions such as coronary and neurodegenerative disease. Symptoms of mercury toxicity at present can go unrecognized due to lack of testing and the difficulty of identifying chronic low-dose exposure symptoms.

In order to further reduce mercury exposure, it is highly recommended that dental amalgams be avoided in favor of other restorative materials such as composites, gold, and porcelain. Humans are also exposed to mercury directly from certain vaccines that contain thimerosal, an EtHg-based preservative. Safety and potential toxicity of thimerosal are hotly debated topics [69, 70]. More research is needed regarding use of thimerosal, along with a simultaneous search for potentially less toxic preservatives.

## 6. Insights and Conclusions

It is important to remember that one indispensable factor in identifying the potential harmful effects of a technology is the nature of the human response. Returning to the Minamata events discussed at the outset of this paper, the mercury that entered Minamata Bay was a by-product of more than 6,000 tons of acetaldehyde produced each year by the Chisso Corporation. No one knew that mercury in any form was responsible for the severe and traumatic health events affecting the local population, even though mercury was used as a catalyst in the industrial process. It was only through a finding made by the team of investigating Japanese physicians led by Masazumi Harada [71] that the involvement of mercury was discovered. Healthy pregnant women, eating their normal diet of local seafood, were inadvertently exposing their unborn children to high doses of MeHg [23]. Neurological and developmental pathologies in these children were caused by transmission of MeHg through both the placenta and breast milk [72]. It is important to recall that the methylmercury causing Minamata disease was produced not on purpose, but as an unintended consequence of an industrial error. “Although mercury was used as a catalyst and a catalyst normally does not change during the chemical process, in this case a reaction other than the target one (i.e., a side reaction) is assumed to have occurred, producing methylmercury that flowed out into the sea mixed with wastewater of the factory” [73, 74].

Let us look for a moment at the language used here. From the point of view of the industry, the production of methylmercury was just a side effect, or “side reaction” of the industrial process. Yet from the point of view of the human and nonhuman population affected by the methylmercury, the “side effect” was in fact the main effect, the only consequence of importance to them. The linguist Benjamin Lee Whorf, who initially worked as a fire insurance investigator, discovered that some industrial accidents had a linguistic factor: fires were caused by lighted matches being thrown into what were seen as “empty gasoline drums”; but the gasoline drums were not in fact empty [75]. Similar linguistic factors may be at work here and in other areas of technology. It would be a profound irony of history if through yet unforeseen multiple synergistic consequences of “side effects” connected with other technologies, such as the fossil fuel economy, industrial farming, nuclear reactors, nuclear weapons, and developments in biowarfare, the entire human species faces an unprecedented extinction event.

Back in 1972, biologist Garret Hardin made this observation:

“*Food chains are only imperfectly known; the extent of biological magnification is imperfectly known; long term, clinical effects on human beings need more study; and we do not know the probability that the whole system of nature will ultimately be disrupted, and human existence imperiled. But it is greater than zero.*” *And he added* “*This is why, now that mercury is a pollutant of the ocean, there is a health hazard in eating tuna and swordfish, which are at a high trophic level, and not much of one in the eating of shrimp. Someday (if we do not mend our ways), even the shrimp will become inedible. Ultimately the algae themselves will be toxic*” [76]. Hardin prophesied his warnings more than forty years ago. His words deserve repeating today, just after the midpoint of the second decade of the 21^st^ century.

## Figures and Tables

**Table 1:** Environmental methylmercury concentrations measured in air, water, and sediments.

Location of mercury	Amount or range of MeHg concentrations	Air, water, and sediment transport	Literature source
Air in Europe	Occurs in picograms/m^3^. Picograms are equivalent to parts per trillion (ppt).	Mercury is transported worldwide, regionally, and locally.	[40]
Water from Sacramento River Basin, CA (where most gold mining took place during 1849–1981)	0.27–2.84 ng/g, which is equivalent to parts per billion (ppb).	Serves as distribution source of Hg via rain, streams, and run-off from lakes and oceans. Hg becomes CH_3_Hg through microbial processes in water.	[41]
Sediments collected from within SFO Bay and at Oakland estuary	SFO Bay samples analyzed 2.3 ppm (dry weight) in SFO Bay's Oakland Estuary.	Hg trapped in aquatic sediment allows sulfate-reducing bacteria to transform elemental Hg to organic CH_3_Hg.	[42]

**Table 2:** Methylmercury concentrations in representative trophic level I organisms.

Trophic level I	Methylmercury (MeHg) concentration	Comments	Literature citation
Primary producers: plants and algae that synthesize their own energy and food	Concentrations are species specific	Beginning of accumulation and concentration into biological systems	[22, 25, 27]
Phytoplankton from Lake Almaden, south of SFO Bay	Range: below 1.50 (the detection limit) to 8.2 ng/g dry weight (ng/g equivalent to ppb)		[47]

**Table 3:** Methylmercury concentrations in representative trophic level II organisms.

Trophic level II	Methylmercury (MeHg) concentration	Comments	Literature citation
Primary Consumers: these are primarily herbivores, filter feeders, small prey fish, etc.	Range: 0.009 to 4.83 ppm	Methylmercury is sequestered in adipose and visceral tissue, and concentrations continue to accumulate in higher trophic levels	[41, 47]
Zooplankton	0.009 ppm		[47]
Clams	0.009 ppm		[48]
Sardines	0.013 ppm		[33]
Cod	0.111 ppm		[49]
Small-prey fish	Range: 1.22–4.83 ppm		[4]

**Table 4:** Methylmercury concentrations in representative trophic level III organisms.

Trophic level III	Methylmercury (MeHg) concentration	Comments	Literature citation
Secondary consumers: carnivores that consume herbivores	Range: 0.1–0.3 ppm	Methylmercury is retained in tissues of organisms. Organisms of trophic level III are consumed by organisms in higher trophic levels IV and V; the toxin continues to accumulate.	[49]
Herring	0.1 ppm		[49]
American lobster	0.1 ppm		[49]
Catfish	0.2 ppm		[49]
Black sea bass	0.2 ppm		[49]
Dragonfly larvae from Golden Gate National Recreation Area, San Francisco	0.3 ppm (mean)		[52]
California clapper rail (eggs)	0.3–0.8 ppm	Anemia, disturbed gait, feather abnormalities, neurological changes, and immunological damage.	[53]

**Table 5:** Methylmercury concentrations in representative trophic level IV organisms.

Trophic level IV	Methylmercury (MeHg) concentration	Comments	Literature citation
Tertiary consumers: carnivores that consume other carnivores	Range: 0.008–1.6 ppm	Mercury toxicity leads to the following reproductive consequences in avian life: eggshells of developing bird embryos are thinner and more fragile, and offspring exhibit decreased appetite and poor development	[55]
Salmon (canned)	0.008 ppm		[49]
Salmon (frozen/fresh)	0.022 ppm		[49]
Halibut	0.241 ppm	Fish are likely to experience reproductive complications due to bioaccumulation of methylmercury in the viscera, which lines the abdomen	[49]
Forster's tern (eggs) sampled from SFBA	0.9–1.6 ppm		[27]

**Table 6:** Methylmercury concentrations in representative trophic level V organisms.

Trophic level V	Methylmercury (CH_3_Hg) concentration	Comments	Literature citation
Apex predators: highest level consumers in food chains	Range: 1.0–6.1 ppm	Organisms in this level contain the highest levels of methylmercury, as MeHg has biomagnified up through the food web. Concentrations are species specific.	[49, 53, 58]
Largemouth bass	6.6 ppm		[53]
Selected shark species	1.0–4.0 ppm		[49]
Three-spined stickleback	Seasonal range: 0.6–1 ppm (dry weight)	Sticklebacks collected from the Alviso Slough, South San Francisco Bay.	[59]

## Data Availability

The data used to support the findings of this study are available from the corresponding author upon request.
